# BiPred: A Bilevel Evolutionary Algorithm for Prediction in Smart Mobility

**DOI:** 10.3390/s18124123

**Published:** 2018-11-24

**Authors:** Jamal Toutouh, Javier Arellano, Enrique Alba

**Affiliations:** Departamento de Lenguajes y Ciencias de la Compuitación, Universidad de Málaga, 29071 Málaga, Spain; jarellanol@lcc.uma.es (J.A.); eat@lcc.uma.es (E.A.)

**Keywords:** smart mobility, road-traffic prediction, dataset reduction, evolutionary algorithms, machine learning

## Abstract

This article develops the design, installation, exploitation, and final utilization of intelligent techniques, hardware, and software for understanding mobility in a modern city. We focus on a smart-campus initiative in the University of Malaga as the scenario for building this cyber–physical system at a low cost, and then present the details of a new proposed evolutionary algorithm used for better training machine-learning techniques: BiPred. We model and solve the task of reducing the size of the dataset used for learning about campus mobility. Our conclusions show an important reduction of the required data to learn mobility patterns by more than 90%, while improving (at the same time) the precision of the predictions of theapplied machine-learning method (up to 15%). All this was done along with the construction of a real system in a city, which hopefully resulted in a very comprehensive work in smart cities using sensors.

## 1. Introduction

From the very first concepts of a city, thousands of years ago, it was clear that mobility inside the city and between cities would be the main domain of development. Today, with most of the world population living in cities (and more to migrate there in the next 50 years) transportation of goods, citizen mobility, and new models of road traffic are continuously appearing. Indeed, the speed at which new challenges in mobility in cities is larger than the services given by city councils and collaborating companies. With the new concept of smart cities [1,2,3,4,5,6], countless new problems have been appearing in mobility [7,8,9], including: parking, optimized routes, car sharing, smart systems in buses, private models of mobility, signaling, lane decisions, social implications of mobility, energy consumption, and environmental implications.

The need for information to take decisions based on the gathered data is today a prominent problem. Even very basic data on mobility are missed most of the time: number of cars per street, origin/destination (OD) matrices, pedestrian behavior, or vehicle types. All are hardly found together, and, indeed, they are very important indicators of the status of the city. Considering that key performance indicators (KPIs) like those are a basic first step to progress toward a smarter city, we can conclude that we are all in trouble. We need initiatives to create real systems to measure KPIs and communicate them to a server for an intelligent decision making. There are some initiatives to advance this knowledge of city traffic roads, at least to gather a minimum set of open data that allow stakeholders to create so-called vertical services. Traditional measurement hardware [10] is expensive, hard to install, and scarcely available, so it has been replaced with new technologies like sensors, cameras, and floating cars [11]. In this context, new services for the mobility department and for citizens appear every year based in the intelligent management of the sensed information [12,13].

In this context, smart mobility based on smart traffic-management systems is a key aspect in the development of smart cities. Intelligent systems able to detect, predict, and efficiently manage different road-traffic situations would endow city officials with powerful tools for a number of new services, such as the reduction of travel time and greenhouse-gas emissions. These systems are all based on accurate traffic monitoring as the first step (as well as historical data) to later allow needed services to be built on top.

As mentioned above, existing traffic-monitoring systems utilize expensive and road-intrusive devices, e.g., loop detectors. Thus, it is paramount to find new ways of having real data from the city at a low cost and in a nonintrusive manner. The present technology allows to obtain very precise road-traffic density measures (e.g., the number and type of vehicles). However, it is unable to individually identify vehicles, and, therefore, cannot obtain information about road-traffic flows (e.g., OD matrices). In fact, technologies based on video license-plate recognition are also used for this purpose. However, this technology presents several drawbacks, the main ones being the high costs of installation and management, and too-high variable accuracy that depends on external situations, such as meteorology. So, an important research question is whether there is any low-cost option to loop sensors and cameras. The constraints to answer this question are important too: low cost, providing rich information, respecting anonymity, and capable of scaling.

Some researchers have proposed systems based on detecting Bluetooth (BT) signals and identifying vehicles and pedestrians by the hardware Media Access Control (MAC) addresses of their devices [14]. This low-cost alternative is experiencing fast development, since it is very cheap and easy to maintain. However, its accuracy depends on the market penetration of the target BT devices (with present low detection rates between 5% and 12%) [15]. Instead, some recent studies have considered Wi-Fi (IEEE 802.11 wireless-based) signals to track pedestrians to overcome the inaccuracy of BT-based sensors because Wi-Fi is very frequently used by citizens [16]. In addition, an increasing number of car manufacturers include wireless access points as part of the standard equipment of their cars.

In this work, we briefly discuss the building and installation of the cyber–physical system that we developed at the campus of the University of Malaga. As a note on the actual goal of this article, the prediction of traffic flow is the first base step for a further service for smart mobility (based on using intelligent systems). We propose the detection of vehicles by capturing the wave signals generated by the smart devices that are located inside them, e.g., on-board units or drivers’ smart devices (e.g., smartphones and tablets).

In short, the proposed cyber–physical system captures information from wireless devices (BT and Wi-Fi) and road-traffic noise. The combined use of the two wireless technologies allows a greater collection of MAC addresses. We gathered (and transmitted to a central server facility) these data for weeks for this article, then made a basic filtering-and-go for its use in this work to better know our streets. We provide a new technique based on coupling an evolutionary algorithm (EA) [17] and machine learning (ML) techniques, that can predict traffic density, while reducing the amount of data required to generate the prediction. This mixture of theory and real practice can be useful for many researchers. In this case, we apply a polynomial regression model as the base ML method, but nothing prevents the extension to other ML methods. Our results indicate that the technique has a very good quality of prediction, based in a bi-level design of algorithms: a structured hierarchical way of using techniques with expected good results.

Therefore, the main contributions of this article are:

- introducing the cyber-physical system installed in the campus of University of Malaga to capture road-traffic information;
- defining the optimization problem of finding a data subset able to accurately describe the knowledge of the whole road-traffic dataset, when it is applied over a given ML method (polynomial fitting in this case);
- and proposing the BiPred approach based on coupling an EA and an ML method, which has been able to reduce the required data to learn mobility patterns in more than 90%, while improving the precision of the predictions up to 15%.

This paper is organized as follows. The next section outlines the related work, since we go for hardware realizations plus data management and intelligent algorithms, we make a short discussion of the most important topics. Section 3 introduces the cyber-physical system allowing us to gather the real data used in this work. Section 4 formally defines the problem addressed in this article. Section 5 presents our proposed Bilevel Predictor (BiPred) algorithm based on coupling EAs and ML. Section 6 shows the carried-out experiments and discusses their encouraging numerical performance. Finally, Section 7 outlines our conclusions and new (more sensible) lines of research after this work.

## 2. Related Work

Since the more innovative part and the focus of the paper (the BiPred algorithm) is a new hybrid-form EA and ML for prediction, we review these domains here. Many works have been carried out with the aim of predicting future events from a set of historical data. The use of time series is maybe the most popular way to carry out analysis of historical data for prediction, so let us start there.

Time series have been widely used in analysis and prediction tasks in many real-world applications, e.g., to analyze economic cycles [18], to determine the relationship between the weather (temperature) and population mortality [19] or air pollution, the weather, and violent crimes [20]. Other authors have applied time series to study data that came from artificial satellites [21]. In our study, we focus on the prediction of road-traffic data to provide future smart-mobility services.

As the amount and complexity of the data has grown, new methodologies for efficient data management are mandatory. Accurate and unbiased estimations of the time-series data produced by modern applications cannot always be achieved using well-known linear techniques, and, thus, the estimation process requires more advanced time-series prediction algorithms.

In recent years, different techniques of Artificial Intelligence (AI), such as neural networks (NN), support vector machines (SVM), pattern recognition (PR), and adhoc heuristics have been used for the analysis of time series and the prediction of events. The NN approaches to time-series prediction have been widely used [4,22].

An NN approach was used to find the appropriate sample rate and window size [23]. The authors used a heuristic method for finding the appropriate sampling rate and embedding dimension to decide the window size. Connor et al. proposed a robust learning algorithm and applied it to recurrent NNs (RNNs) [24]. This algorithm is based on filtering out layers (noise) from the data and then estimating parameters from the filtered data. To show the need for robust RNNs, the authors compared the predictive ability of least squares-estimated RNNs on synthetic data and on the Puget Power Electric Demand time series.

Another tool that has been successfully used in recent years to improve and expand the field of prediction is SVM [25,26,27]. In the cited works, the capacity of SVM to operate with noisy data was clearly shown, hence the importance of this technique for real predictions.

The use of PR is then paramount, and is addressed in many cases by using time series [28,29]. Time-series clustering has been shown effective for various domains, but it needs a deep extension of concepts if we want to deal with clustering in non-Euclidean spaces. Specifically, a cluster prototype needs to be calculated. In Reference [29], authors defined an optimal prototype as an optimization problem, and proposed a local-search algorithm. The authors experimentally compared different time-series clustering methods, and found that the proposed prototype with accumulative clustering (by *K*-Means) provided the best clustering accuracy. Additionally, a method for clustering time series exists based on their structural features, as proposed in Reference [30]. Unlike other approaches, this method does not cluster point values using a distance metric, but rather it clusters data based on global features extracted from the time series themselves.

After reviewing all these previous works, the conclusion about the state of the art is that techniques are very varied if we want an accurate and efficient treatment of the sensed data, with a clear presence of ML in all of it. It is also well-known that, in ML, the quality and amount of the available data influences the final results. In our case, the quality of the prediction for a complex system, like vehicles moving in city. At a higher level, these related works told us that there is a niche for new improvements in smart mobility.

EAs have been seen as a tool to address ML by a number of authors for the last three decades [31]. Specifically, genetic algorithms (GA) have been successfully used to define a number of different types of genetic-based machine-learning systems, e.g., GA-based pattern recognition [32] or neuroevolution (i.e., GA-based NN) [33,34]. However, we go for a proposal that applies EAs to enhance an ML prediction method by reducing the data required to create a predictor (more efficient and accurate learning).

Focusing on the use of EAs to improve ML methods, several authors have faced the feature selection problem [35]. An early approach applied a GA to identify and select the best subset of features to be used by a rule induction system [36]. The same authors compared their GA-based approach with a greedy-like search method (specifically designed to address this feature selection problem) [37]. They concluded that the GA was able to provide more competitive results since the greedy-like search had a tendency to get trapped on local minima.

Recently, Amira Sayed et al. evaluated a GA-based feature-selection method for anomaly detection. The authors used several feature-selection techniques, e.g., principal-component analysis (PCA), sequential floating, and correlation-based feature selection [38]. Automatic Feature Subset Selection using GA (AFSGA) was introduced to automatically identify the required features to compute representative clusters, while reducing the computational cost of the used clustering method [39]. A GA was also applied to find a subset of features to solve the problem of face recognition and a generic model for 3D facial expressions [40]. The results showed that this approach provides good results in addressing face modeling.

On the one hand, these previous studies principally focus on feature selection, i.e., selecting the minimum subset of variables that allow to represent all data in a unique way. On the other hand, in the present article, we deal with the problem of selecting the minimum subset of data (road-traffic measures registered by our sensors) that will characterize a time series with the same trend as the original dataset. Therefore, an important difference with feature selection lies in the fact that we want to find a subset of data of which the mathematical distribution is similar to the distribution of the original dataset (without losing generality). As shown in this paper, the model obtained from the selected subset of road-traffic measures can predict road-traffic behavior based on the number of cars registered by the sensors.

## 3. Building a Cyber–Physical System

The goal of this article is to propose a new algorithm for the intelligent analysis of data coming from vehicles in city streets. However, we have many added values to this analysis, because we have built the whole system and exploited it, and this is related and of interest to other researchers who want to examine real situations, from the lab to the streets, at a low cost. Thus, we take some time in this section to introduce the Internet of Things (IoT) system that we have developed, which is used to capture the data used by the intelligent BiPred algorithm. An early version of this system was previously introduced in Reference [41].

This paper describes an initial step of our work to make our university campus a Smart Campus. Before retrieving the very first data, we had to build and operate what is called a cyber–physical system. In such a system, we deal with specialized hardware and software to enable the monitoring of streets and elaborate a set of files full of numerical data that we can trust to finally start addressing our true goal.

Though the system itself is not our target, the effort in building and using it do have implications on the amount of data and their quality. This is the first article in a family of articles that foster low-cost technology, light IoT devices, and managing software. We want to show the research community in smart cities and intelligent systems that there is huge potential to be exploited in the coming years at a reasonable cost, with no need for companies or interactions with city managers, simply because a university campus is a microcosm of a city where we can make our real tests.

Our cyber–physical system has several components:

- A set of IoT devices (sensors), to detect Wi-Fi and Bluetooth signals and to measure environmental noise directly from the streets of the city in the campus. We also built the sensors (even if we usually perform research in algorithms) just to prove that this can be done at a low cost, and to have final total control on the data being produced.
- A wireless communication system spanning our (large) campus, to connect IoT devices with a central server where the info is gathered and stored.
- A server interacting with the sensors, where specialized software is used to produce useful knowledge from the raw data collected by the sensors.

In future works, we plan to fully describe this system, which will need a few separate papers focused in computer and software engineering, and other multidisciplinary domains. In this paper, we just wanted to point out the existence of the system to better explain the domain in which we are moving; quite different from downloading a benchmark problem from a website in a one-minute operation. Even if open data start to be available on the Internet, we are still in a very initial phase of developments in smart systems.

All this is mentioned to support our claim of realism in the data that we are managing and in the aim of making predictions: the system producing the data is real and is operating right now, the information is real from the vehicles, and the benefits of predictions are real for the city. Even if we go here for an algorithm and its evaluation, we believe in the power of added values in research since they inspire and guide other researchers.

First, we describe the IoT devices used to collect the data that we later used in our experiments, and second, we defined how they integrate into the global cyber–physical system in terms of communications and utilization.

### 3.1. Sensor Description

**Figure 1:**
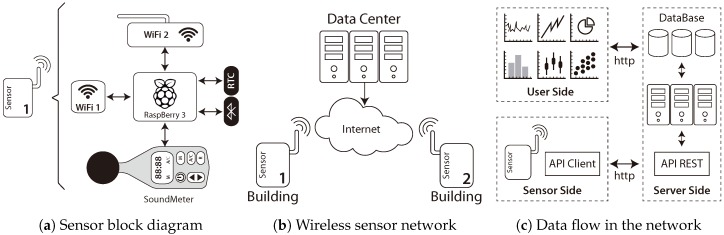
Different elements and views of the road-sensor system [41].

In order to overcome the high cost and limitations of traditional traffic data-collection methods, the presented sensor utilizes broadly used low-cost technologies. Figure 1a shows the block diagram of the designed sensor. As can be seen, the sensor is composed of three wireless interfaces: two for wireless-network connections (Wi-Fi 1 and Wi-Fi 2) and one for Bluetooth, then a Real-Time Clock (RTC) and a noise–sound meter.

These devices constitute the core of the system since they are responsible for the measurement of the data captured by the sensor and later processed. All these devices are controlled by a Raspberry Pi 3 (Raspberry Pi 3 specs. https://www.raspberrypi.org/products/raspberry-pi-3-model-b/) small computer, which is responsible for filtering and temporarily storing the data generated. In summary, the sensor constantly collects raw data coming from Wi-Fi 2, Bluetooth, and the sound meter by storing locally.

To carry out the monitoring of road traffic, we used the architecture shown in Figure 1b. A set of sensors (at least two) is needed to collect the road-traffic data and flows of vehicles. Periodically (by using the Wi-Fi 1 interface), such data are transferred through the Internet to the server, which is located in a data center, where it is aggregated and analyzed.

In order to carry out the transfer of data between the sensors and the data center, a software client/server architecture was designed (see Figure 1c); this one implements a web service that establishes the protocols for the correct exchange of data.

### 3.2. Sensor Operation

The developed sensor retrieves three types of data: noise level, and MAC addresses of the nearby Bluetooth and Wi-Fi devices.

The first type of data is obtained by using a sound meter, model GM1356, YH-THINKING part number BCBI10216, which measures the level of noise twice per second. The noise sensor communicates with the central processing unit by using a USB connection. Readings are stored in a database on the server. In order to make a noise map, the equivalent sound is calculated. Equivalent sound pressure level, i.e., $L_{eq}$ expresses the mean of the energy sound perceived by an individual (measured in decibels (dB) in an interval of time [42]). Thus, $L_{eq}$ represents the level of pressure that would have been produced by a constant noise with the same energy as the noise actually perceived during the same time interval.

Bluetooth networks (commonly referred to as piconets) use a master/slave model to control when and where devices can send data; thus, to detect a Bluetooth device, the sensor must be continually open and in discovering mode. Any device with Bluetooth enabled is continually sending data packets on the search for other Bluetooth devices. These data packets are also read by our sensor to obtain the MAC addresses in order to identify the devices. This information, together with the exact time, is stored in the server for further analysis. Finally, it is important to note that the effective range of a Bluetooth link varies due to propagation conditions, material coverage, production sample variations, antenna configurations and battery conditions. In our initial tests, it has been determined that the range of coverage of our sensor for detection of Bluetooth devices is around ten meters.

Wi-Fi technology is used to detect additional devices even when they have no Bluetooth enabled. Wi-Fi devices that implement the 802.11 protocol constantly transmit beacons that include their MAC address as well as other important information. The Wi-Fi interface of the sensor is operating in promiscuous mode to intercept all the beacons in the air transmitted by other Wi-Fi devices. Once a beacon is captured by the Wi-Fi interface, the MAC address and signal strength are extracted and stored. As the signal strength generated by Wi-Fi devices is higher than that of the Bluetooth, the Wi-Fi effective range of coverage is longer. For this reason, the sensor detects Wi-Fi devices at longer distances.

The combination of these three types of data (sound noise, Bluetooth, and Wi-Fi devices) allows us to provide a global knowledge of the status of road traffic: we sense data, convert it into aggregated information and then build knowledge out of it. In simple words, the current number of the devices detected by a giving sensor is a measurement of road-traffic density. The accuracy of this metric can be improved by using the noise level returned by this sensor.

Indeed, road-traffic flows can be inferred by determining the trajectories of the devices followed throughout our sensor network in a given time window. Evaluating MAC addresses allows a fine evaluation of the flows since they contain the type of device (e.g., cellphone, wearable, vehicle, etc.). Moreover, further analysis of this information can be carried out to get the most used roads, the faster routes, the most noisy places, etc.

## 4. Traffic-Monitoring System Design Optimization

The main purpose of developing the proposed system is to allow city managers to evaluate the current traffic situation and to predict road traffic. This type of prediction assistance in decision making improves the use of road-traffic resources in order to, among others, reduce the number of traffic jams during rush hours and avoid dangerous situations.

The type of prediction proposed here presents several challenges. One of the most salient is the accurate treatment of the large amount of data collected by the sensor system to generate useful knowledge, since most of ML methods would suffer from overfitting issues. Therefore, it is important to find the most efficient way to analyze the generated data with the aim of avoiding these issues without losing precision in the generated knowledge or prediction.

Out of all the different ML techniques, we have selected one with low computational complexity: polynomial fitting (PF) or regression predictor [43]. PF consists of finding a polynomial function that has the best fit to a series of data points (i.e., data distribution). In this case, our data distribution is defined by the number of vehicles (number of different MAC addresses), which represents the road-traffic density captured in a given period of time. This polynomial is used to characterize and later predict road traffic in the location of the city where the sensor is installed.

The main idea behind BiPred is to find a subset of the sensed data to apply a ML method (PF in our case), which could be used to accurately describe the knowledge of the whole dataset regarding road traffic, while avoiding, or at least limiting, the negative impact of overfitting.

The selection of such an optimal subset of data, which in fact are sensed data during some specifics periods of time during the day, would also improve the efficiency of the process of retrieving the sensed data and predicting. This is mainly because future iterations over the ML method (PF) to improve predictions would use just the data sensed during these specific period of times and not the whole dataset. In turn, the sensors would be configured to more efficiently use computation and communication resources during such periods of time in order to ensure accurate measurements.

**Figure 2:**
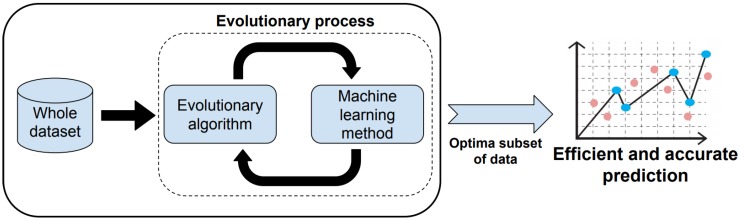
BiPred methodology.

Therefore, BiPred presents a two-level predictor: first, it applies an intelligent and automatic method based on coupling an EA and ML to select the best subset of data, that is, the smaller one that allows to obtain an efficient and accurate predictor. Figure 2 summarizes the methodology applied to obtain efficient predictions by using BiPred.

The search of the best subset of all the sensed data during a day is not easy since there is an unaffordable number of possible combinations that should be analyzed, depending on how the sensed data are grouped or split up. If all the sensed data are grouped in data blocks that combine the data sensed during *m* min, the number of possible subsets of data (NPS) is computed according to Equation (1). Thus, if we group the data in blocks of five minutes, there are $2^{288}$ possible combinations to be analyzed.

(1) $$NPS=2^{ss},\;where\;ss=\frac{24\;h\times 60\;\min/h}{m}$$

With such a huge number of possible solutions (subsets of data), exact and enumerative methods are not applicable for solving the underlying search and optimization problem of finding the best configuration because they require critically long execution times to perform the search, and because we are far from having a traditional analytical model. In this context, EAs are a promising approach to find accurate subsets of the sensed data to apply ML in reasonable execution times [17].

## 5. Evolutionary Algorithm for Efficient Prediction

In this section, we describe the evolutionary method used to address BiPred in order to build a bilevel predictor for road traffic in the streets.

### 5.1. Evolutionary Algorithms

EAs are nondeterministic methods that mimic the Darwinian evolutionary process of species in nature to address search, optimization, and other similar problems [44,45]. In the last three decades, EAs have been successfully applied for addressing hard-to-solve problems underlying many real and complex applications.

Algorithm 1 describes the generic schema of an EA. Basically, it iteratively applies stochastic operators on a set of solutions (individuals) that belong to a solution set named population (*P*) in order to improve their quality according to the objective of the problem, which is measured according to a given fitness value. Each iteration is called generation.

**Algorithm 1** Generic schema for an evolutionary algorithm (EA).
1:*t*← 0                {generation counter}2:**initialize**(*P*(0))3:**while** not stopcriterion **do**4:   **evaluate**(*P*(*t*))5:   parents ← **selection**(*P*(*t*))6:   offspring ← **crossover**(parents)7:   offspring ← **mutation**(offspring)8:   *P*(*t* + 1) ← **replacement**(offspring, *P*(*t*))9:   *t* ← *t* + 110:
**end while**
11:**return** best solution ever found


Every individual in the population encodes a candidate solution for the problem. The initial population is generated by a random method or by using a specific heuristic for the problem (line 2 in Algorithm 1). An evaluation function associates a fitness value to every individual, indicating its suitability to the problem (line 4).

The search process is guided by a probabilistic technique of selecting the best individuals (parents and generated offspring) according to their quality (line 5). Iteratively, solutions evolve by the probabilistic application of variation operators (lines 6–7). Thus, new solutions are generated by using the crossover operator that recombines parts from two individuals (parents) and the application of random changes (mutations) in individuals. The stopping criterion usually involves a fixed computational effort (number of generations, number of fitness evaluations, or execution time), a quality threshold on the best fitness value, or the detection of a stagnation situation.

Specific policies are used to select the groups of individuals to recombine the selection method and to determine which new individuals are inserted in the population in each new generation (the replacement criterion). Finally, the EA returns the best solution ever found in the iterative process, taking into account the fitness function.

In this study, we apply a variant of EAs based on a GA [45] to devise our efficient predictor, BiPred. In the following subsections, we describe the main components of this evolutionary method: the solution encoding, the evolutionary operators, and the evaluation of the objective function.

### 5.2. Solution Encoding

In order to address BiPred, the whole sensed data is grouped in clusters of a given length of time (in this study, we group the data in clusters of 5 min). Thus, BiPred decides which of these groups of data are taken into account to generate the fitting polynomial to predict road traffic over the 24 h of a day.

In the proposed BiPred, solutions are represented as vectors of binary values (bit strings), having a size dependent on the length of the time used to group the sensed data. In this case, the length of the solution vector is 288 (i.e., number of 5 min blocks in 24 h). The first position of the vector represents the first 5 min of the day (from 0:00:00 h to 0:04:59 h), the second represents the data sensed from 0:05:00 h to 0:09:59 h), and so on. The last position of the solution represents the data obtained from 23:55:00 h to 23:59:59 h. Each position on the vector contains the information about whether such a data block is considered (1), or not (0), to compute the fitting polynomial. Thus, BiPred searches for the efficient solution (subset of data) over 2^288^ possible solutions.

**Figure 3:**
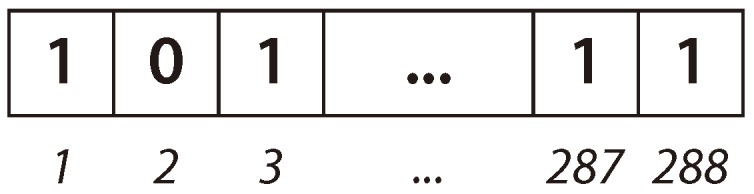
Representation of an individual solution used by BiPred.

Figure 3 shows an example of an individual in which the bits in positions 1, 3, 287, and 288 are ones. Thus, this tentative solution (subset of data) includes the data sensed from 0:00:00 h to 0:04:59 h (block 1), from 0:10:00 h to 0:14:59 h (block 3), and from 23:50:00 h to 23:59:59 h (blocks 287 and 288), respectively.

### 5.3. Evolutionary Operators

In the following, we describe the applied evolutionary operators to generate diversity during the search: initialization, crossover, and mutation.

#### 5.3.1. Initialization

In this study, the individuals of the initial population are randomly generated. Each bit that is part of the bit strings that represent the solutions have the same probability to be initialized to one or zero. This strategy allows the EA to start the evolutionary search from a subspace of solutions that use about half of the sensed data to make efficient predictions.

#### 5.3.2. Crossover

A specific crossover operator, 12-Hour Recombination (12 h), has been designed in order to address BiPred. This operator is applied with a probability $p_{C}$ to recombine genetic information of two parents in order to generate two new solutions or offspring. On the basis that solutions are made by 24 blocks of 12 bits that represent the bit masks in each hour of the day (24 × 12 = 288), i.e., each block of these 12 bits represents the data collected from XX:00:00 h to XX:59:59 h. The 12 h operator is applied over two parents ($P_{1}$ and $P_{2}$) that exchange with each other 12 blocks of an hour to create the offspring ($O_{1}$ and $O_{2}$). These 12 blocks are randomly chosen from each one of the 24 h.

**Figure 4:**
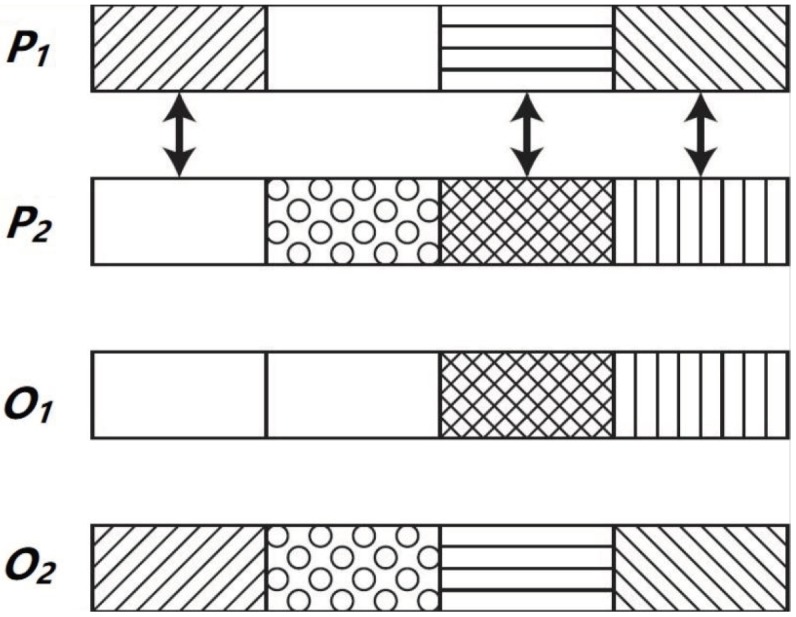
Crossover operation applied by the EA of BiPred.

Figure 4 summarizes how this crossover operator is applied over two parents to obtain the two offspring individuals. In this case, it is shown partially the individuals (just four blocks of data). The two parents ($P_{1}$ and $P_{2}$) exchange three data blocks of an hour to generate the offspring ($O_{1}$ and $O_{2}$).

#### 5.3.3. Mutation

**Figure 5:**
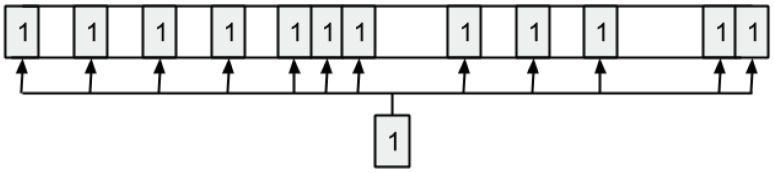
Mutation operation applied by the EA of BiPred.

We have devised an adhoc operator in order to provide enough diversity to the search process, avoiding our evolutionary approach to get stuck in a specific region of the search space. The mutation operator probabilistically spreads 12 new data blocks through the solution or removes 12 data blocks over the whole solution. Figure 5 shows an example of how this mutation operator is applied to generate diversity. In this example, it adds 12 new blocks through the solution.

### 5.4. Evaluation of the Objective Function

A fitness function is defined to evaluate the generated solutions and guide the search during the evolutionary process carried out by BiPred. This function takes into account two different metrics: Mean Squared Error (MSE) over all the sensed data, and the required size of the dataset used to compute the polynomial fitting. The first metric is the MSE of the fitting polynomial computed with the data represented (masked) by the current solution pol(s), when it is applied over all the sensed data. The second metric is evaluated in terms of a ratio of the data used by the current solution, which is the number of ones in the solution over the size of the bit string that represents a solution (size(s)). Thus, fitness function f(s) is used to evaluate the solutions is computed according to Equation (2). This fitness function defines a minimization problem (minimizing both the MSE and the size of the used data).

(2) $$f\left(s\right)=MSE\left(pol\left(s\right)\right)+\frac{\sum_{i=0}^{i<size(s)}s\left(i\right)}{size(s)}$$

## 6. Experimental Analysis

This section describes the experimental evaluation of the proposed evolutionary approach to address the BiPred. Experimental analysis was carried out in a Magni-Core cluster with 48 cores at 2.2 GHz, with 48 GB RAM. In the following subsections, we describe the problem instances, EA parameterization experiments, optimization numerical results, and the validation of the obtained polynomials computed.

### 6.1. Problem Instances

The BiPred problem was addressed over real-world instances. These proposed problem instances are defined by using real-world data captured by one of the sensors devised by us that is located in the campus of the University of Malaga.

**Figure 6:**
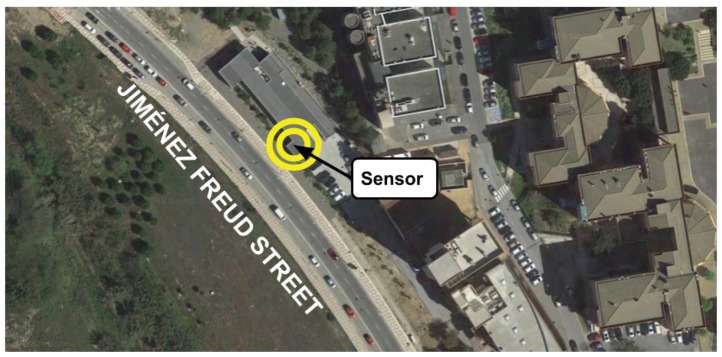
Location of the sensor (two circles) that captured the data used in this study.

Specifically, we defined the problem instances according to the collected data (road-traffic density in terms of number of vehicles sensed in a given time) by a sensor located in the main front of the Laser Laboratory of the University of Malaga. This building is placed in the middle of Jiménez Freud Street (Málaga, Spain) (see Figure 6). This street is one of the main entrances to the university campus, it is 315 m long, and it has a three-lane road in which vehicles move with speeds between 30 and 50 km/h. The road in this street has no traffic lights, but two crosswalks are located at the corners.

**Figure 7:**
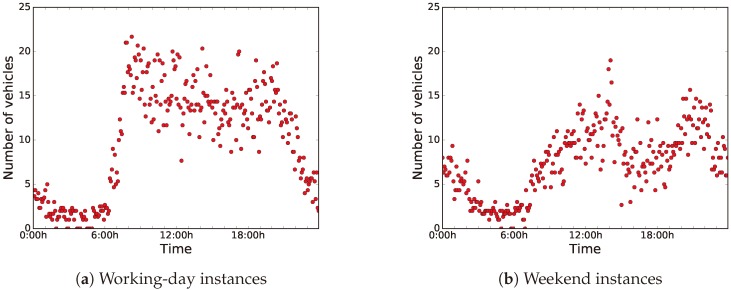
Clustered data regarding number of vehicles sensed every five minutes.

The data were collected over 61 days, from May 1, 2018 to June 30, 2018. Figure 7 shows the distributions of the sensed data during working days (from Monday to Friday) and weekends (Saturday and Sunday). For ease of analysis, we have clustered the data in groups of data blocks of five minutes each, and we have used the centroids for the computations.

As can be seen in Figure 7a,b, there is a clear difference between the distribution of the data of working days and weekends. On the one hand, during working days, road-traffic density is higher and it stays regular during working hours. On the other hand, on weekends, road-traffic density is quite higher during night-time, because people usually go out at night, since they are free from work the next day.

Thus, we have taken into account two different use cases or instances, working days and weekends, in order to develop an accurate predictor. In this article, we applied BiPred to these two different instances (working days and weekends).

In order to validate the efficiency of BiPred, we defined the base polynomial that best fits the distribution of all the data obtained by the sensor for both instances. Thus, we applied the PF method but, as stated before, nothing prevents the use of the evolutionary methodology presented here to be used with other ML methods.

As the accuracy (in terms of MSE) of this fitting method is highly dependent on the degree of the used polynomial, we have selected the best-suited degree according to the elbow method [46]. Broadly speaking, the elbow method is a visual method to obtain the most promising value from a line chart where a change in the slope looks like the elbow of an arm. Thus, this method looks at the percentage of variance explained as a function of the degree of the polynomial. In our experiments, we have evaluated polynomials with degrees from one to eight.

**Figure 8:**
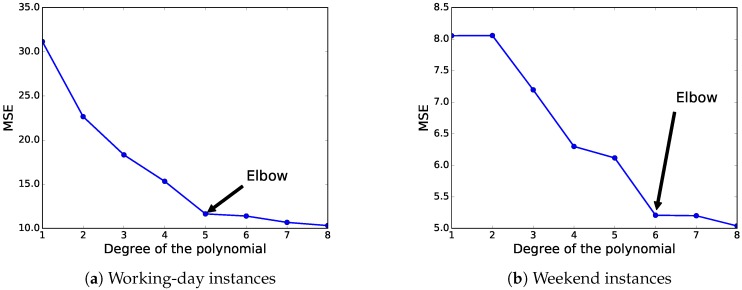
Polynomial fitting results (Mean Squared Error, MSE) by using different polynomial degrees.

**Figure 9:**
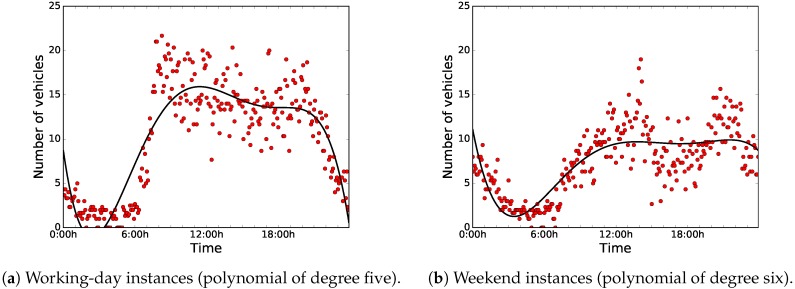
Data distributions and selected polynomials.

Figure 8 illustrates the results in terms of MSE when applying different degrees of the polynomial fitting method to the whole dataset, and the elbow-method results. For the working-day instances, we can see that the polynomial of the fifth degree is the best choice to be used in this predictor, according to the elbow method. Regarding weekend instances, the elbow method states that the most competitive result in terms of MSE is obtained by the polynomial of the sixth degree. The selected polynomials obtained an MSE of 11.641 and 5.208 for the working-day and weekend instances, respectively. Figure 9 shows the data distributions and the selected base polynomials according to the elbow method for both instances.

### 6.2. EA Parameter Settings

In order to find the best values for the EA parameters, a set of experiments have been carried out. For crossover $p_{C}$ and mutation $p_{M}$ probabilities to use, we considered the same, following four tentative values: 0.2, 0.4, 0.6, and 0.8. Therefore, we evaluated 16 (4 × 4 = 16) different parameterizations.

As BiPred uses a nondeterministic EA, we ran each algorithm 30 times for each problem instance. Table 1 and Table 2 summarize the results in terms of the final fitness computed for each configuration and for working-day and weekend instances, respectively. These tables show average, standard deviation, minimum, median, and maximum fitness values for the 30 independent runs.

For working-day instances, the best (lowest) results were obtained by the parameterizations of the algorithm with the highest crossover probabilities (see Table 1). On the one hand, the configuration that computed the lowest average, minimum, and median is the one with $p_{C}$ = 0.8 and $p_{M}$ = 0.6. On the other hand, the configuration $p_{C}$ = 0.8 and $p_{M}$ = 0.2 obtained the worst fitness value (11.235,516).

The results in Table 2 show that, in general, the best performance is also obtained by the configurations with $p_{C}$ = 0.8 for the the weekend instances. However, the best result (minimum) was computed by the $p_{C}$ = 0.6 and $p_{M}$ = 0.8 algorithm parameterization. The lowest average, median, and maximum final fitness values were obtained by the configuration with $p_{C}$ = 0.8 and $p_{M}$ = 0.6.

We applied a statistical procedure [47] to select the best configuration. First, we applied the Kolmogorov–Smirnov statistical test to evaluate whether the result distributions follow normal distribution or not. As the experimental results did not follow normal distributions, we applied Friedman Rank nonparametric tests to rank the configurations regarding the final fitness computed.

**Figure 10:**
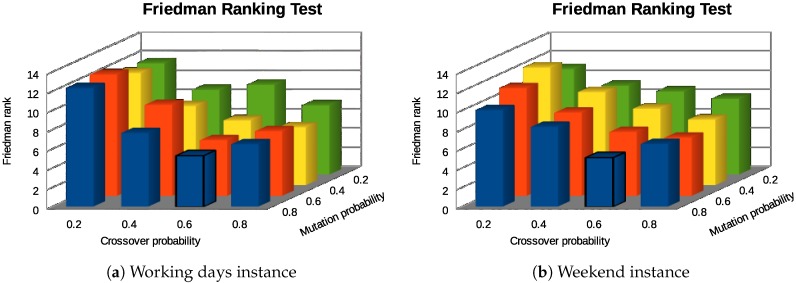
Friedman Rank test results for parameterization experiments.

The bar diagrams in Figure 10 summarize the Friedman Rank test results. As expected, the best parameterization of BiPred for both instances is the one with $p_{C}$ = 0.8 and $p_{M}$ = 0.6. The results were obtained with *p*-values ≪ 0.001 for both instances. Therefore, the significance of these results was higher than 99%.

### 6.3. Numerical Results

This subsection summarizes and analyzes the main results of the experimental evaluation of the proposed BiPred algorithm applied over the two real-world instances defined here: working days and weekends. The results shown are those corresponding to 100 independent runs performed on each instance. According to the results of the previous subsection, the algorithm was set to perform 500 generations with a population of 20 individuals, $p_{C}$ = 0.8, and $p_{M}$ = 0.6.

Table 3 reports the final fitness values and the number of data blocks of the computed solutions by our BiPred on both problem instances. It includes the average, standard deviation, best (minimum), median, and worst (maximum) values computed over the 100 independent executions performed.

The results in Table 3 show that the proposed BiPred was able to significantly reduce the needed number of blocks of data to compute accurate polynomials fitted to the whole dataset (i.e., reduced MSE) for both instances. The best solutions found so far provided the lowest MSE (11.09 and 4.34) and required the smallest amount of data blocks (17 and 11).

**Figure 11:**
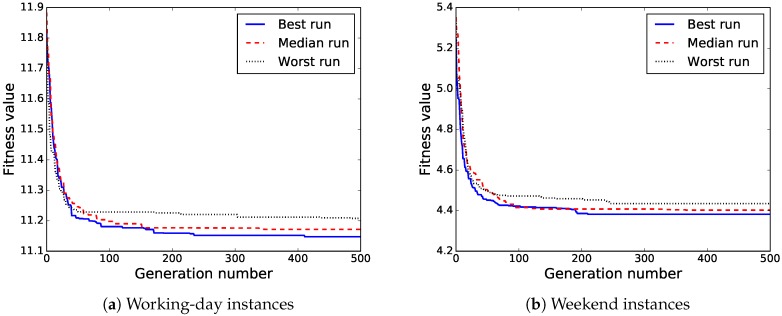
Fitness evolution through the best, median, and worst independent run.

In order to provide better insight into the evolutionary process of BiPred, Figure 11a,b show the evolution of the fitness value of the best solution found for the independent runs that computed the best, median, and worst solutions for working days and weekend instances, respectively. It can be observed that BiPred is able to compute accurate solutions quickly, with good convergence. However, we notice that for the last generations the algorithm performs differently in each of the instances. On the one hand, in working-day instances, the best found solution was improved during the last 100 generations of the best run. On the other hand, in the weekend instances, the best computed solution was not modified during the same last generations.

**Figure 12:**
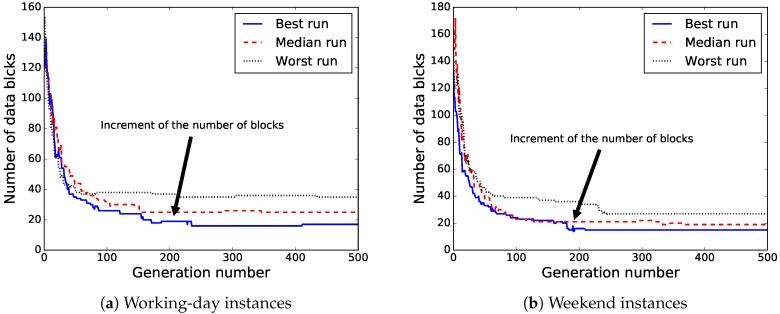
Evolution of the number of blocks through best, median, and worst independent run.

Figure 12 illustrates the evolution of the number of data blocks used by the best solution found for the independent runs that computed the best, median, and worst solutions for both instances. Thus, we can see that there are improvements in the fitness of the solutions with a larger number of data blocks (see Figure 12). This is mainly due to our approach being able to improve the MSE by redistributing the data blocks in such a way that reduces the fitness values even as the number of data blocks increases.

### 6.4. Results Validation

In order to validate the results, we compare the polynomials provided by the proposed BiPred method against two different ones: first, the one computed taking into account the whole dataset, which is named base polynomial, and second, the one computed by applying the K-fold cross-validation method [48,49,50].

Table 4 reports the improvements achieved by BiPred-computed polynomials over the base one in terms of MSE and number of data blocks. The results clearly state that BiPred is able to improve the prediction computed by taking into account all the sensed data in both scenarios studied here. As to the analysis of working days, BiPred could reduce the MSE by up to 4.57% while reducing the required data blocks by more than 94.09%. When analyzing the improvements in the weekend scenario, they were even more competitive. The best solution reduced MSE by 16.15% while using less than 4% of the sensed data. Thus, the numerical results demonstrate that accurate predictions may be carried out by using a subset of fine-selected data.

**Figure 13:**
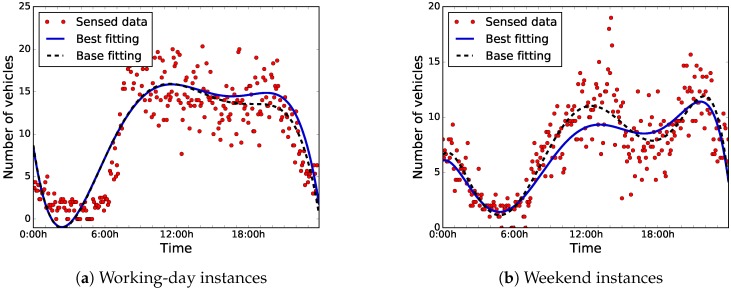
Best-found fitting polynomials.

Figure 13 shows the sensed data, the best-found, and the base-fitting polynomials for both instances. It is remarkable that there are no large differences between the best-fitting polynomials to the base ones for situations with low road-traffic densities (from 0:00 h to 6:00 h).

Cross-validation is commonly used to compare and select a model for a given predictive modeling problem. The goal of cross-validation is to estimate the expected level of fit of a model to a dataset that is independent of the data that were used to train the model. In this case, we compared BiPred with a nonexhaustive cross-validation method, i.e., K-fold cross-validation.

**Figure 14:**
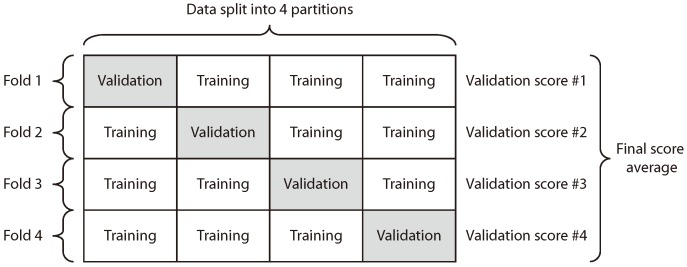
K-fold cross-validation method (*k* = 4).

K-Fold provides train/test indices to split data in training and testing sets. It splits the dataset into *k* consecutive folds (see Figure 14). For the evaluation of the algorithm proposed in this work, we chose the K-Fold method with *k* = 4 as a cross-evaluation scheme. This method was chosen due it is useful when the performance of the model shows significant variance based on the train–test split.

Table 5 presents the MSE obtained by the best polynomial found by BiPred and by the polynomial computed by using K-fold cross-validation. The results in the table shows that our method provides better fitted polynomials to the dataset since the MSE obtained by the proposed algorithm is lesser than the one computed by K-fold.

## 7. Conclusions and Future Work

In this article, we have shown an interesting contribution in the application of intelligent systems (EAs and ML) for data analysis of vehicle flows in modern cities. The cyber–physical system capturing these data was completely built by the authors of this work in an effort to show that there is a cheap and sizeable way of having a positive impact on the quality of life of citizens.

The main goal of this paper was not the system itself, but the application of the proposed approach, BiPred, in which an EA is coupled with an ML method to perform accurate and efficient predictions. In this case, BiPred has been applied to predict road-traffic density in terms of the number of vehicles during the day in real-world locations in the city of Málaga (Spain).

BiPred has shown good quality in numerical analyses so as to even allow the city council to make informed decisions on mobility. The many difficulties in preparing data (because of missing values, noisy information, etc.) and the intelligence needed in the techniques to evaluate the quality of a solution for an EA (both in the algorithm’s design and its implementation) have been dealt with in this paper so as to show readers a direction to follow in similar future works.

As for the numerical findings, we have been able to deal with different types of days (working days and weekends); we reduced the required data to model the predictor by more than 90%, compared to the whole dataset, and we improved the accuracy of the predictions by more than 4% and 15% during working days and weekends, respectively.

Nowadays, we are working on extending the wireless sensor network with more sensors in order to obtain information from other points of our city. We are also analyzing the use of other intelligent systems and algorithms (multiobjective EAs, particle-swarm optimization, NNs, etc.) to address three important challenges that arise from the use of our sensors to provide smart-mobility solutions: first, obtaining road-traffic flows from thecaptured data (OD matrices); second, aggregating sensed environmental noise to improve the knowledge of the current road-traffic status; and third, designing the most efficient wireless sensor network by selecting the type and the location of the sensors. Finally, we are also cleaning the data files and constructing a website to open up this work to the research community.

## Figures and Tables

**Table 1:** Parameterization results for working-day instances—final fitness values.

$p_{C}$	$p_{M}$	Average ± Stdev.	Best (Minimum)	Median	Worst (Maximum)
0.2	0.2	11.191973 ± 0.015237	11.157625	11.190797	11.227597
0.2	0.4	11.181100 ± 0.014414	11.146663	11.182200	11.214257
0.2	0.6	11.183086 ± 0.017350	11.154759	11.176878	11.225440
0.2	0.8	11.177069 ± 0.014018	11.151867	11.178053	11.216309
0.4	0.2	11.191484 ± 0.016479	11.157764	11.189754	11.228747
0.4	0.4	11.179017 ± 0.013470	11.151626	11.178168	11.206704
0.4	0.6	11.174310 ± 0.012717	11.148484	11.174039	11.204799
0.4	0.8	11.173517 ± 0.016201	11.150723	11.170938	11.209305
0.6	0.2	11.194164 ± 0.015219	11.167866	11.190731	11.228337
0.6	0.4	11.183630 ± 0.012320	11.160233	11.183055	11.205162
0.6	0.6	11.173385 ± 0.010619	11.154660	11.171707	11.196588
0.6	0.8	11.173963 ± 0.013862	11.150161	11.174719	11.208556
0.8	0.2	11.194699 ± 0.014590	11.171123	11.192513	11.235516
0.8	0.4	11.177789 ± 0.009689	11.159919	11.178769	11.196324
0.8	0.6	11.169286 ± 0.012064	11.146485	11.170294	11.196625
0.8	0.8	11.174837 ± 0.011625	11.155679	11.172889	11.203807

**Table 2:** Parameterization results for weekend instances—final fitness values.

$p_{C}$	$p_{M}$	Average ± Stdev.	Best (Minimum)	Median	Worst (Maximum)
0.2	0.2	4.421868 ± 0.018194	4.395552	4.416327	4.469087
0.2	0.4	4.413287 ± 0.013906	4.386546	4.411646	4.440232
0.2	0.6	4.413090 ± 0.012833	4.384903	4.411577	4.441905
0.2	0.8	4.410482 ± 0.018562	4.379673	4.405531	4.450899
0.4	0.2	4.421549 ± 0.011650	4.400080	4.421174	4.444894
0.4	0.4	4.417434 ± 0.016713	4.387659	4.414765	4.473782
0.4	0.6	4.408158 ± 0.014741	4.381670	4.409609	4.438559
0.4	0.8	4.407861 ± 0.013233	4.386163	4.404579	4.446310
0.6	0.2	4.419767 ± 0.012144	4.398379	4.418265	4.449240
0.6	0.4	4.412095 ± 0.012753	4.375144	4.414329	4.431851
0.6	0.6	4.405849 ± 0.011345	4.382822	4.406479	4.424693
0.6	0.8	4.403845 ± 0.011990	4.374438	4.404383	4.422966
0.8	0.2	4.416216 ± 0.009905	4.393292	4.416547	4.436500
0.8	0.4	4.411127 ± 0.009889	4.398651	4.408431	4.433397
0.8	0.6	4.401493 ± 0.008271	4.385474	4.400677	4.417022
0.8	0.8	4.404763 ± 0.013277	4.381001	4.402021	4.432461
0.8	0.8	4.404763 ± 0.013277	4.381001	4.402021	4.432461

**Table 3:** Numerical results over 100 independent runs.

		Average ± Stdev.	Best (minimum)	Median	Worst (maximum)
Working days	Fitness	11.173597 ± 0.012356	11.145738	11.172169	11.207736
	MSE	11.134800 ± 0.012313	11.086710	11.133376	11.168820
	# data blocks	24.380000 ± 3.754411	17	24	35
Weekend	Fitness	4.403659 ± 0.009841	4.380879	4.402977	4.434717
	MSE	4.388368 ± 0.009806	4.342684	4.387688	4.419318
	# data blocks	19.440000 ± 2.677013	11	19	27

**Table 4:** Numerical improvements over base polynomials.

		Average	Best (Minimum)	Median	Worst (Maximum)
Working days	MSE	4.348429%	4.571501%	4.360652%	4.056177%
	# data blocks	91.534722%	94.097222%	91.666667%	87.847222%
Weekend	MSE	15.737932%	16.154674%	15.750990%	15.143659%
	# data blocks	93.250001%	96.180556%	93.402778%	90.625000%

**Table 5:** MSE comparison between polynomials computed by the BiPred and K-fold methods.

	BiPred MSE	K-Fold MSE
Working days	11.08	11.22
Weekend days	4.34	8.40
